# Skin-tone discrimination by Whites and Africans is associated with the acculturation of African immigrants in Norway

**DOI:** 10.1371/journal.pone.0209084

**Published:** 2018-12-13

**Authors:** Jonas R. Kunst, Esther N. Phillibert

**Affiliations:** 1 Department of Psychology, University of Oslo, Oslo, Norway; 2 Department of Psychology, Yale University, New Haven, Connecticut, United States of America; Brown University, UNITED STATES

## Abstract

It is well-established that experiences of discrimination influence immigrants’ acculturation. Yet, whereas a large body of research has demonstrated the role of discrimination by members of the dominant societal group, surprisingly little is known about how being discriminated by members of one’s own group relates to the way immigrants acculturate. With a sample of 162 African first- and second-generation immigrants living in Norway, the present research investigated the relationship between both types of discrimination, acculturation and psychological well-being. It did so, focusing on discrimination based on one’s skin tone, a type of discrimination Africans can experience from White as well as African individuals. Results showed that skin-tone discrimination by Whites was associated with a lower host culture orientation. By contrast, skin-tone discrimination by Africans was associated with a lower heritage culture orientation. Mediation analyses showed that the positive relationship of skin-tone discrimination by Whites and Africans with life satisfaction was mediated by a lower host and heritage culture orientation respectively. This indirect relationship did not reach significance with self-esteem as dependent variable. Participants’ actual skin tone was unrelated to experiences of skin-tone discrimination. We discuss our results in light of previous research and highlight potential limitations.

## Introduction

When immigrants move from one cultural sphere to another, they face the question of how they want to relate to their heritage culture they moved from, and the culture of the host country they moved to [1–3]. While most immigrants prefer to both maintain their heritage culture and to adopt the host society’s culture [4, 5], this choice does not take place in a vacuum but is influenced by attitudes and behaviors in society [6, 7]. One of the most established factors predicting immigrants’ acculturation is the experience of discrimination from members of the dominant societal group, typically predicting less involvement in the host culture [4, 5, 8]. However, immigrants experience and have to navigate attitudes and behaviors not only of the dominant societal group but also of members from their own ethnic groups [9]. Because this has received little attention in previous research, we in this study investigated how discrimination by the dominant societal group as well as by member of one’s own broader ethnic group relate to immigrants’ acculturation orientations and psychological well-being. We did so with a sample of African immigrants living in Norway and focused on discrimination based on one’s skin tone, which is a form of discrimination Africans can experience from both groups.

Skin-tone discrimination has far-reaching historical roots. During European colonialism and slavery, the White ruling class enforced systems that “rewarded those who emulated whiteness culturally, ideologically, economically, and even aesthetically” [10] (p. 239). And to the present date, people often hold implicit associations that “dark skin represents savagery, irrationality, ugliness, and inferiority” whereas whiteness represents “civility, rationality, beauty, and superiority” [10] (p. 238); also see [11] for recent evidence. Indeed, people tend to prefer lighter skin tone in terms of dating and marriage and generally associate it with more positive traits than darker skin tone; and importantly, these preferences are sometimes found also among groups with typically darker skin tone (i.e., people of African descent; see [12] for a comprehensive review; also see [13]). For instance, within the African American community, individuals with lighter skin tone disproportionally marry individuals who also have relatively light skin, a phenomenon referred to as “complexion homogamy” [14]. It has further been argued that these processes are incentivized by a system of rewards and penalties exerted by the dominant societal group [15], at least in countries with a history of slavery such as the U.S.

Hence, as a consequence, certain groups may experience skin-tone discrimination both from the out-group and the in-group [16]. This can sometimes have fatal consequences. In addition to leading to more negative evaluations and prejudice [17], the more “Afrocentric” (i.e., a combination of darker skin tone and prototypical physiognomy) people of African descent are judged to look, the harsher punishment they receive in the criminal justice system, including a heightened likelihood to receive the death penalty [18, 19] (also see [20] for evidence with other ethnic groups).

In previous research, skin tone has been assessed in various ways. Whereas using a spectrometer allows for the most objective assessment with little measurement error (for an example, see [21]), it is seldom used in social scientific research, possibly due to high equipment cost and often low availability. The arguably next best method involves skin-tone ratings by an external investigator on a continuous scale. For instance, the 11-point NIS skin color scale [22] is often used to let evaluators rate participants’ skin tone or to let participants do it themselves. The scale shows individuals a series of pictures of hands that are identical except for the hands’ skin tone that increases in darkness as scores increase from 1 to 11 on the scale. The scale has been validated and shown to be highly related to more objective measures of skin tone such as spectrophotometer reflectance measurements [23].

Building on this measurement method, we used another variant to assess skin tone in the present research, addressing one of the NIS scale’s limitations. Because its 11-point format limits the measure’s precision and potential response variation, we used an analog visual scale that ranged from 0 to 100 instead. Specifically, skin tone was rated on a full visual spectrum from very dark (#200E04, scored as “0” on the scale) to very light (#D29A68, scored as “100” on the scale). Moreover, given that participants’ self-reported skin tone and assessments by external raters often not fully overlap [24], we assessed both. Importantly, given that we had not validated our skin tone scale previously, we let a second external rater assess the skin tone of some of the participants to test for interrater reliability.

Whereas the negative effects of skin-tone discrimination on occupational outcomes and health are well-documented [12, 25], little is known about the specific effects on immigrants’ acculturation experience. A recent study demonstrated that members of dominant societal groups in the West tend to prefer light-skinned to dark-skinned immigrants because they believe that light-skinned immigrants are more likely to assimilate to the dominant culture [26]. Here, we tested how skin-tone discrimination is related to the actual acculturation orientations of immigrants, using a sample of Africans living in Norway.

While the majority-White country Norway used to be ethnically relatively homogeneous before the Second World War, it is now ethnically diverse. Currently, about 1 in 5 Norwegians have an immigration background [27]. Of the total Norwegian population, 127,155 or 2.4% have an African background, making it the second largest non-European group. Only little research has focused on African immigrants’ experience of discrimination in Norway and the scarce available research is mostly qualitative. Available studies suggest that many people of African descent experience explicit and more subtle forms of racism in Norway (e.g., [28, 29]).

Based on the finding that immigrants who experience ethnic discrimination by the dominant societal group often show lower levels of engagement in the national sphere [4, 5, 8], we expected that skin-tone discrimination by Whites would be related to a lower host culture orientation also among African immigrants in Norway. By contrast, given that migrants generally tend to distance themselves from spheres that discriminate them [8], we expected that skin-tone discrimination by other Africans would be related to a lower heritage culture orientation. To the best of our knowledge, no research has investigated the link between these variables so far.

A main reason for why immigrants in response to discrimination reduce their engagement in the host culture or their heritage culture may simply be that they want to avoid future experiences of discrimination. Yet, research on liking and reciprocity may provide further insights into the underlying mechanism. We tend to like people more who like us because we perceive them to have benevolent intentions [30]. Conversely, negative experiences and resulting negative future expectations may reduce our willingness to engage in contact and interactions [31]. This process can, thus, explain why immigrants after experiences of discrimination may retract themselves from the cultural spheres in which they were discriminated.

Importantly, we expected both heritage and host culture adoption, in turn, to be related to more psychological well-being in form of higher life satisfaction and self-esteem [32, 33]. Although not tested directly in the present research, one reason for why high engagement (as compared to low engagement) in both dimensions is generally theorized and empirically shown to predict higher psychological adaptation, is that it can bring social support from the respective groups and bring competence to navigate their respective cultural spheres [34, 35]. Therefore, host culture orientation was expected to mediate the relationship between skin-tone discrimination by Whites on psychological well-being, while heritage culture orientation was expected to mediate the relationship between skin-tone discrimination by Africans on psychological well-being.

## Materials and methods

### Participants

In total, 162 participants took part in the study satisfying sample size recommendations by Wang and Wang [36] and a 10/1 ratio of participants to observed variables in path analyses [37]. Descriptive information about the sample is presented in Table 1. Gender was relatively equally distributed, and after coding one value (“40,000”) as missing, participants were on average young adults. The majority of participants were first-generation immigrants (i.e., born outside Norway), whereas a smaller portion were second-generation immigrants (i.e., born in Norway). According to data from Statistics Norway, the percentage of first-generation immigrants from Africa in the sample (see Table 1) is higher than the actual percentage in the population (62.5%) [27].

**Table 1:** Descriptive information.

Variable		Estimate
Age *M (SD)*		29.56 (10.33)
Female in %		48.1
First-generation Immigrants in %		82.9
Second-generation Immigrants in %		15.9
Grown up in Norway in %		44.5
Parents from African Country in %		94.4
Ethnic Background in %		
	Somalia	30.5
	Burundi	12.2
	Eritrea	12.2
	Ethiopia	6.1
	Democratic Republic of Congo	5.5
	Ghana	4.3
	Rwanda	4.3
	Sudan	3.7
	Morocco	3.0
	Tanzania	2.4
	Cameroon	1.8
	Gambia	1.8
	Nigeria	1.8
	Angola	1.2
	Republic of Congo	1.2
	Guinea	1.2
	Kenya	1.2
	Algeria	0.6
	Botswana	0.6
	Mauritania	0.6
	Sierra Leone	0.6
	South Africa	0.6
Highest Completed Education in %		
	Elementary School	11.7
	High School	46.3
	Lower College/University Degree	14.8
	Higher College/University Degree	14.8
Occupation in %		
	Employed	45.7
	Students	43.8
	Unemployed	4.3
	Other	5.6
Average Income of all participants in *NOK (USD)*		354,433 (~ 44,000)
Average Income of employed participants in *NOK (USD)*		474,312 (~ 58,000)

*Note*. Missing percentages are due to missing values.

Almost half of the participants reported to have grown up in Norway. In terms of their families’ countries of origin, participants reported backgrounds from 22 African countries. The most frequent were Somalia, Burundi, Eritrea, and Ethiopia. The vast majority of participants reported that both their parents also were from an African country.

Finally, in terms of their socio-economic background, most participants had either completed high school or a university degree, and were currently employed or students. Asked about their income, participants on average earned below the average salary of all employees in Norway (531,720 NOK [38]). However, focusing only on the subsection of participants who reported to be employed, this difference was much smaller (see Table 1).

### Procedure

Participants were recruited on public commuter streets, during leisure activities and in community centers for a study on “Integration and inclusion: The Experience of Africans in Norway.” The surveys were available in English and Norwegian (bokmål). To ensure correct translations, all questions were forward-back translated from English into Norwegian by bilingual teams. Participants completed the surveys on tablet computers and data were recorded on the platform provided by the survey company Qualtrics. All participants received chocolate candy for participation. The study was approved by the Institutional Review Board of the Department of Psychology at the University of Oslo (Nr: 1841452).

### Instruments

Participants completed the following items on 7-point Likert scales, ranging from 1 (*strongly disagree*) to 7 (*strongly agree*), unless stated otherwise. In addition to the measures presented here, a measure of social dominance orientation [39] was also included in the survey but not analyzed. This data is publicly available to interested researchers at https://osf.io/m2c8t/?view_only=f9beb509df4140dd98a780863d7b2e00. Please note that the ethnicity of participants has been omitted from this publicly-available dataset to warrant anonymity.

#### Discrimination

We adopted a measure by Uzogara et al. [16] to assess whether participants felt that they were treated better or worse because of the shade of their skin tone by (a) White people and (b) other Africans. Response options were 1 (*a lot better*), 2 (*somewhat better*), 3 (*no different*), 4 (*somewhat worse*), 5 (*a lot worse*). Hence, participants completed this question twice, once measuring their perceived treatment from White people and once from African people. The order of the items was random.

#### Acculturation orientations

When operationalizing acculturation, researchers have to be cautious in determining the cultural dimensions they want to assess, which, in turn, requires them to identify “bounded and appropriately labeled groups” [40] (p. 978). The Norwegian nation state only relatively recently became a country of immigration and its White, native majority population is commonly defined as ‘ethnically Norwegian’ [41]. Hence, we used the term “Norwegian” in combination with different cultural domains to assess host culture adoption in the present research. Moreover, given that we collected a multi-ethnic sample, we in terms of heritage culture maintenance did not specify a specific African ethnic group but rather assessed acculturation in terms of participants’ “ethnic group.”

Specifically, based on Berry et al. [4], participants completed each seven items measuring maintenance of their heritage culture (i.e., “heritage culture orientation”) and adoption of the dominant Norwegian culture (i.e., “host culture orientation”) in the seven domains culture, customs, values, traditions, way of living, friends, and identity. For instance, in terms of their heritage culture orientation, participants were asked to rate their agreement with the statement, “I prefer to maintain the cultural customs of my ethnic group,” and equivalently in terms of their host culture orientation, “I prefer to adopt Norwegian customs.” The reliability of both scales was satisfactory (heritage culture orientation: α = .87; host culture orientation: α = .87).

#### Life satisfaction

Using the satisfaction with life scale [42], participants indicated their agreement with five items such as “In most ways my life is close to my ideal.” The scale had acceptable reliability (α = .79).

#### Self-esteem

Using the Rosenberg self-esteem scale [43], participants indicated their agreement with ten items such as “I feel that I am a person of worth, at least on an equal plane with others.” The scale had acceptable reliability (α = .78).

#### Skin tone

**Fig 1:**
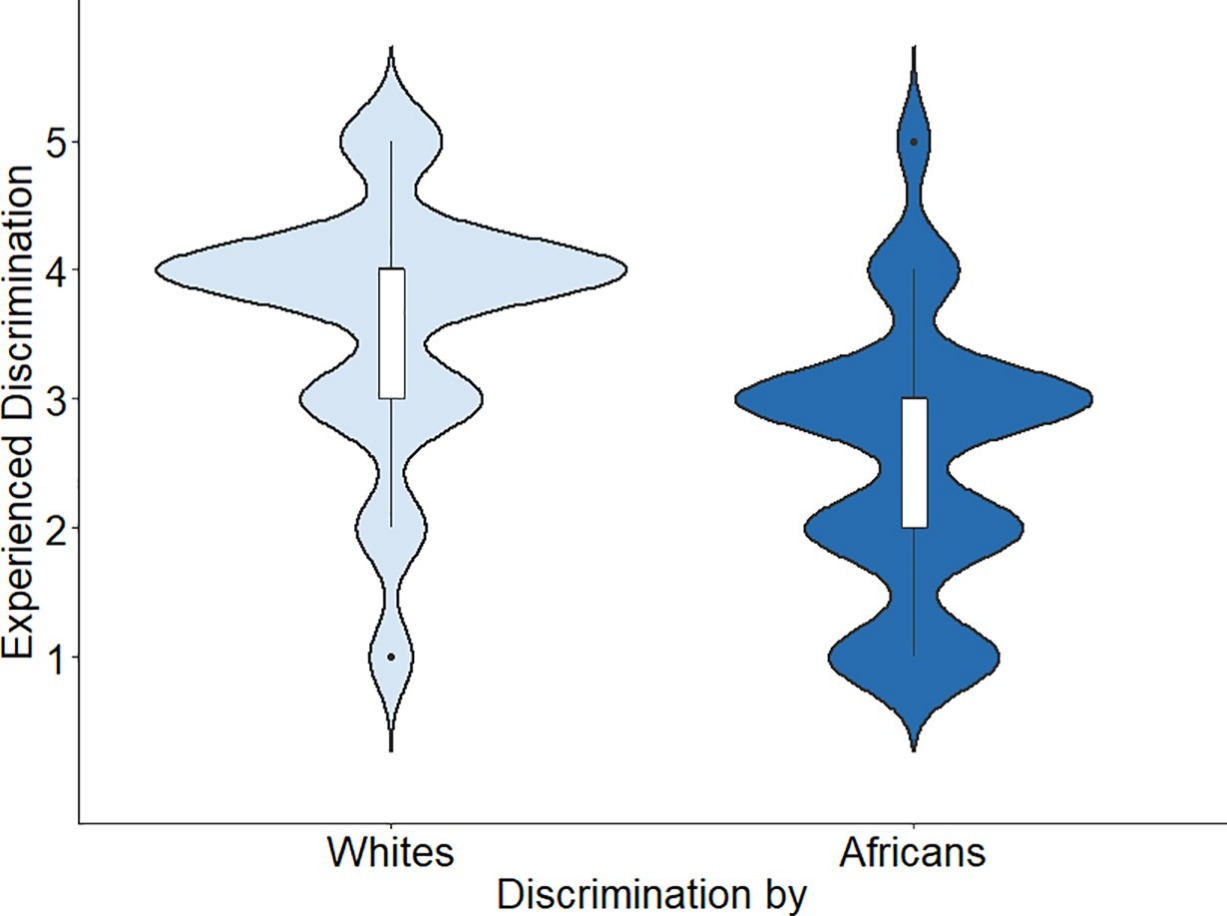
Discrimination experiences. Individuals experienced more skin-tone discrimination by Whites than by Africans. Response distributions and boxplots are displayed. Different colors are used for presentational purposes. The y-axis shows the full range of possible values (1–5).

On a visual analog scale (see Fig 1), participant indicated their own skin tone, with ratings ranging from 0 (*reflecting the darkest color #200E04*) to 100 (*reflecting the lightest color #D29A68*). In addition, to get a more objective estimate, the individual administering the surveys measured participants’ skin tone by comparing the scale to the skin tone of the underarm of participants. To test the reliability of this estimate, a second coder rated the skin tone of ten participants. The inter-rater correlation was very high, *r*(8) = .96; *p* < .001. The self-reported skin tone and the skin tone measured by the rater correlated moderately, see Table 2.

**Table 2:** Means, standard deviations and correlations for the main study variables.

	*M*	*SD*	2.		3.		4.		5.		6.		7.		8.	
1. Discrimination by Whites	3.60	.97	.12		-.24	**	-.04		-.09		.09		.06		-.03	
2. Discrimination by Africans	2.53	1.04			-.06		-.21	**	-.06		-.01		-.08		-.07	
3. Host Culture Orientation	4.32	1.25					.11		.20	**	.06		-.12		.01	
4. Heritage Culture Orientation	4.97	1.22							.24	**	.16	*	-.01		-.07	
5. Life Satisfaction	4.83	1.23									.26	**	.18	*	.14	
6. Self Esteem	5.43	.97											.12		-.04	
7. Self-Reported Skin Tone^a^	53.44	27.25													.65	***
8. Skin Tone Reported by Rater^a^	55.01	22.48														

^a^Higher values mean lighter skin tone.

*p < .05.

**p < .01.

***p < .001.

## Results

### Skin tone and discrimination

**Fig 2:**
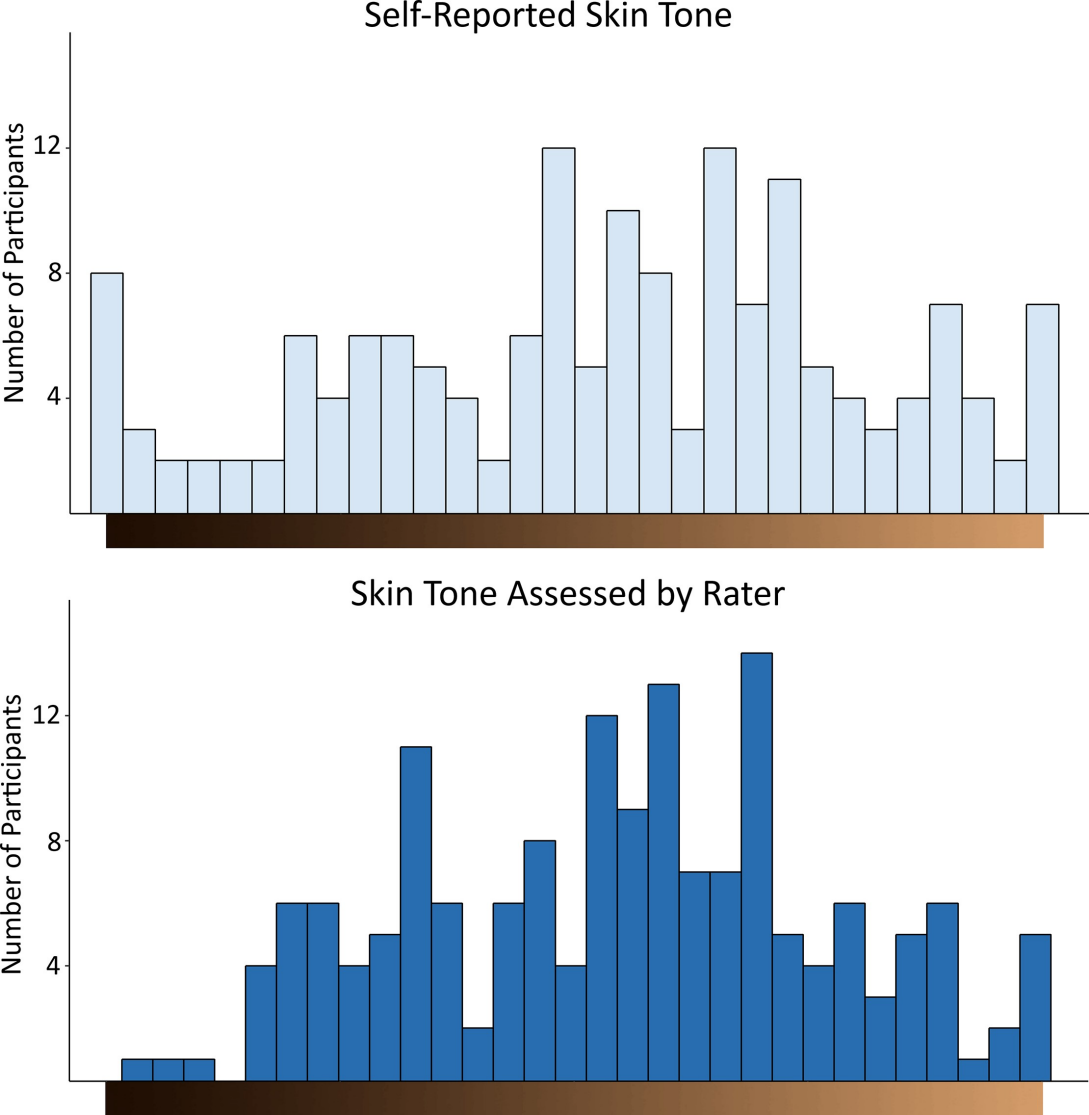
Skin-tone distribution. Distribution of self-reported skin tone (upper panel) and skin tone assessed by the rater (lower panel). The color spectrum displayed on the x-axis represents the exact visual analog scale used to assess participants’ skin-color. As displayed, the scale was anchored by the colors #200E04 (equal to a “0” response on the scale) and D29A68 (equal to a “100” response on the scale).

As displayed in Fig 1, experiences of being discriminated by Whites due to one’s skin tone were higher than experiences of being discriminated by Africans, *t*(161) = 10.12, *p* < .001, *d* = -.82. On average, participants scored on the middle of the skin tone self-report measure, *M* = 53.44, *SD* = 27.25, and the response distribution was relatively normal, skewness = -.26, kurtosis = -.74, see Fig 2. The same was the case for skin tone assessed by the rater, *M* = 55.01, *SD* = 22.48, skewness = -.05, kurtosis = -.70, that appeared even closer to a normal distribution. Yet, regardless of which measurement approach was used, individuals’ skin tone was unrelated to the degree of discrimination they experienced from Whites and Africans (see Table 2). Because previous research from the U.S. found that both “too dark” and “too light” African American individuals experience the most discrimination [16], we also tested for quadratic relationships in a regression. However, also these relationships remained non-significant, *ps* > .166.

Of the demographic variables, age was associated with self-reported skin tone, *r*(159) = -.20; *p* = .012, with older individuals reporting darker skin tone. In addition, women had a lighter skin tone both assessed as self-report, *M*_*Women*_ = 62.77, *SD*_*Women*_ = 24.03 vs. *M*_*Men*_ = 44.79, *SD*_*Men*_ = 27.34, *t*(160) = 4.43, *p* < .001, *d* = .70, and as assessed by the rater, *M*_*Women*_ = 60.69, *SD*_*Women*_ = 19.54 vs. *M*_*Men*_ = 50.83, *SD*_*Men*_ = 23.23, *t*(158.48) = 2.93, *p* = .004, *d* = .46. Women, as compared to men, were also more likely to report being discriminated by White people, *M*_*Women*_ = 3.77, *SD*_*Women*_ = .76 vs. *M*_*Men*_ = 3.47, *SD*_*Men*_ = 1.09, *t*(146.69) = 2.04, *p* = .043, *d* = .32. First-generation immigrants had darker skin tone assessed as self-report, *M*_*First-Generation*_ = 51.32, *SD*_*First-Generation*_ = 26.83 vs. *M*_*Second-Generation*_ = 64.54, *SD*_*Second-Generation*_ = 27.24, *t*(160) = 2.30, *p* = .023, *d* = -.49, and assessed by the rater, *M*_*First-Generation*_ = 54.03, *SD*_*First-Generation*_ = 21.08 vs. *M*_*Second-Generation*_ = 63.69, *SD*_*Second-Generation*_ = 25.37, *t*(160) = 2.07, *p* = .040, *d* = -.41, than second-generation immigrants had. Finally, individuals who were raised in Norway reported more discrimination based on their skin tone from Whites, *M*_*Raised in Norway*_ = 3.81, *SD*_*Raised in Norway*_ = .72 vs. *M*_*Raised outside Norway*_ = 3.45, *SD*_*Raised outside Norway*_ = 1.08, *t*(152.25) = 2.48, *p* = .014, *d* = .39, and reported to have lighter skin tone than those who were raised outside Norway, *M*_*Raised in Norway*_ = 58.70, *SD*_*Raised in Norway*_ = 24.48 vs. *M*_*Raised outside Norway*_ = 49.13, *SD*_*Raised outside Norway*_ = 28.03, *t*(160) = 2.25, *p* = .026, *d* = .36.

### Acculturation

In terms of acculturation orientations, participants on average scored clearly above the midpoint of the heritage culture orientation scale, *M* = 4.97, *SD* = 1.22, and slightly above the midpoint of the host culture orientation scale, *M* = 4.32, *SD* = 1.25. As expected, skin-tone discrimination by Whites was negatively correlated with host culture orientation, whereas skin-tone discrimination by Africans was negatively correlated with heritage culture orientation (see Table 2). Heritage culture orientation was related to more life satisfaction and self-esteem, and host culture orientation to more life satisfaction.

Of the demographic variables, age was negatively related to heritage culture orientation, *r*(159) = -.30; *p* < .001, with older individuals maintaining their heritage culture less. Moreover, women reported a lower host culture orientation than men did, *M*_*Women*_ = 4.02, *SD*_*Women*_ = 1.26 vs. *M*_*Men*_ = 4.60, *SD*_*Men*_ = 1.18, *t*(160) = -3.04, *p* = .003, *d* = -.48. First-generation immigrants had a stronger host culture orientation than second-generation immigrants had, *M*_*First-Generation*_ = 4.44, *SD*_*First-Generation*_ = 1.19 vs. *M*_*Second-Generation*_ = 3.65, *SD*_*Second-Generation*_ = 1.39, *t*(160) = -3.02, *p* = .003, *d* = .61. Finally, individuals who were raised in Norway reported a lower host culture orientation than those who were raised outside Norway, *M*_*Raised in Norway*_ = 4.08, *SD*_*Raised in Norway*_ = 1.33 vs. *M*_*Raised outside Norway*_ = 4.51, *SD*_*Raised outside Norway*_ = 1.16, *t*(160) = -2.24, *p* = .026, *d* = -.34.

For exploratory reasons, we used the midpoint-split procedure [44] to categorize participants into the four acculturation strategies first outlined by Berry [1]. Using this procedure, 43.3% of the participants were integrated (i.e., had high scores on both dimensions), 31.1% separated (i.e., had a low host culture score but a high heritage culture score), 12.8% assimilated (i.e., had a high host culture score but a low heritage culture score), and 11.6% were marginalized (i.e., had low scores on both dimensions). However, participants did not differ in terms of self-esteem, *F*(3, 157) = 1.43, *p =* .237, discrimination by Whites, *F*(3, 157) = 1.91, *p =* .131, discrimination by Africans, *F*(3, 158) = 2.33, *p =* .076, self-reported skin tone, *F*(3, 158) = 1.07, *p =* .366, and externally-rated skin tone, *F*(3, 158) = 1.73, *p =* .163, depending on the acculturation strategy they were categorized into. Only one difference for life satisfaction was observed, *F*(3, 158) = 2.85, *p =* .039. Bonferroni-corrected post-hoc comparisons here showed that integrated individuals showed higher life satisfaction, *M* = 5.08, *SD* = 1.20, than marginalized individuals, *M* = 4.21, *SD* = 1.31, *p* = .035. Assimilated, *M* = 4.70, *SD* = 1.01, and separated individuals, *M* = 4.75, *SD* = 1.25, did not differ from the other groups and no additional significant differences were observed, *ps* > .598.

### Test of the path model

Next, we set out to test our predictions with a path model. In this model, discrimination by White individuals was expected to be related to a lower host culture orientation, whereas discrimination by Africans was expected to be related to a lower heritage culture orientation. Both orientations were, in turn, expected to predict higher self-esteem and life satisfaction. Based on the results reported above, we also controlled for the effect of age on the proposed mediator heritage culture orientation, and for the effects of participants’ gender, the place where they were raised and their generational status on host culture orientation. The model showed very close fit to the data, χ^2^(19) = 13.15, *p* = .831, *Root Mean Square Error of Approximation* (*RMSEA*) < .001, 95% CI [.000, .042], *Comparative Fit Index* (*CFI*) = 1.00, *Standardized Root Mean Square Residual* (*sRMR*) = .034. Two participants were automatically excluded from analysis given missing data patterns.

**Fig 3:**
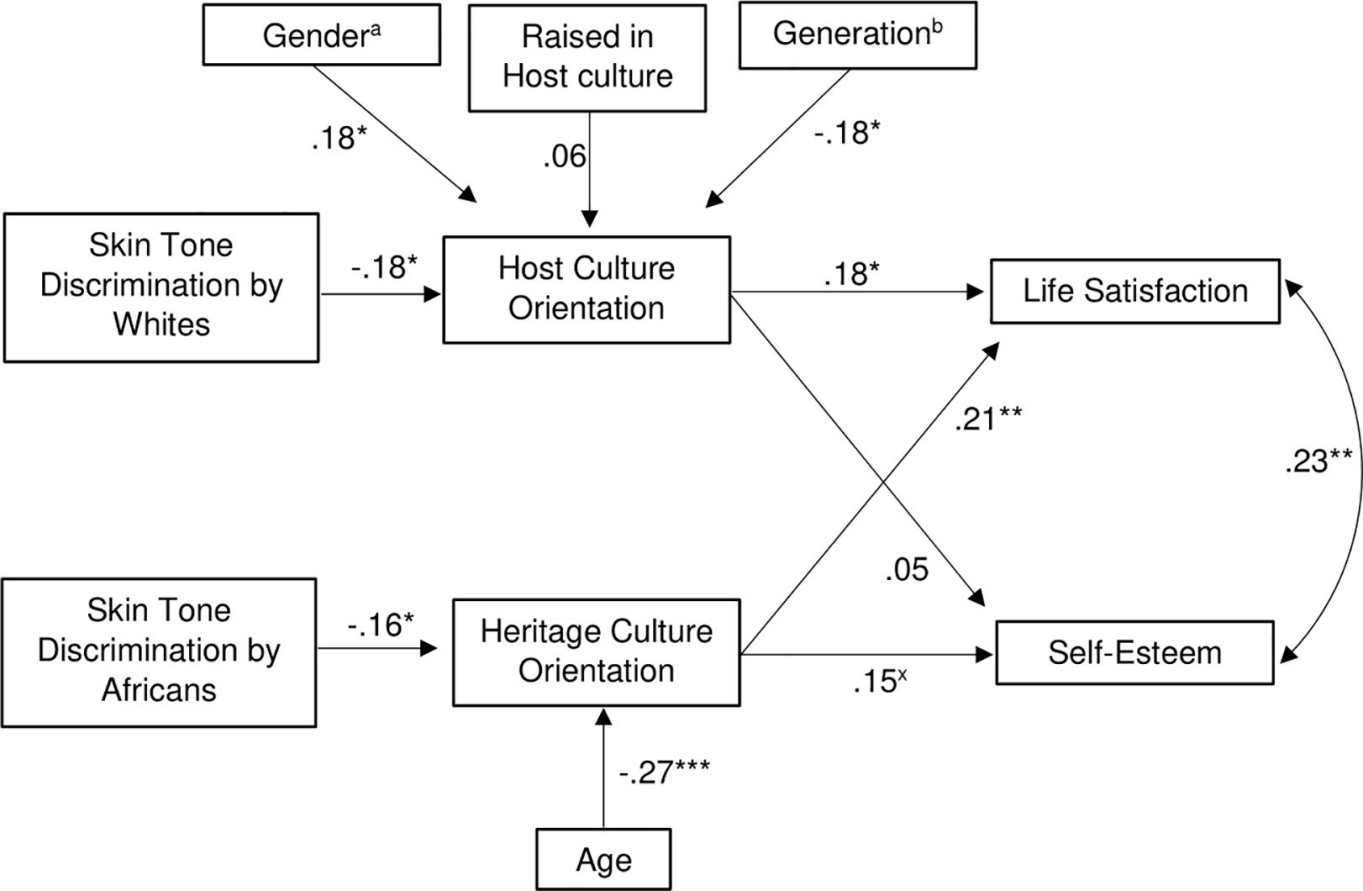
Path model. ^a^coded as 0 = female, 1 = male. ^b^coded as 0 = first generation, 1 = second generation. ^x^*p* = .053, *p < .05, **p < .01, ***p < .001.

In the model (see Fig 3), skin-tone discrimination by Whites predicted a lower host culture orientation, whereas skin-tone discrimination by Africans predicted a lower heritage culture orientation. Of these orientations, host culture orientation predicted higher life satisfaction, while heritage culture orientation predicted higher life satisfaction and, marginally significantly, higher self-esteem. Bootstrapping with 5,000 random re-samples showed that the indirect effect of discrimination by Africans on life satisfaction that was mediated by heritage culture orientation was significant, *B* = -.04, 95% CI [-.107, -.002]. Also, the indirect effect of discrimination by Whites on life satisfaction that was mediated by host culture orientation was significant, *B* = -.04, 95% CI [-.117, -.005]. By contrast, neither the indirect effect of discrimination by Africans on self-esteem that was mediated by heritage culture orientation, *B* = -.02, 95% CI [-.073, .001], nor the indirect effect of discrimination by Whites on self-esteem that was mediated by host culture orientation, *B* = -.01, 95% CI [-.054, .021], reached significance.

## Discussion

Whereas previous research has shown that discrimination by members of the dominant societal group affects how immigrants acculturate, there is a lack of research on the additional role that discrimination from one’s own ethnic group may play. In a sample of African immigrants living in Norway, we therefore investigated the role of both factors, focusing on skin-tone discrimination. As predicted and consistent with previous research [4, 5, 8], skin-tone discrimination by Whites was related to lower levels of host culture orientation. Importantly, at the same time, skin-tone discrimination by other Africans was related to lower heritage culture orientation. Both orientations were in turn related to higher life satisfaction. Accordingly, skin-tone discrimination by Whites and Africans were indirectly related to less life satisfaction, mediated by lower host culture orientation and heritage culture orientation respectively.

Previous research has shown that immigrants’ acculturation is influenced by acculturation *expectations* from both the dominant majority group and by members of their own ethnic groups [9]. While our findings are correlational and do not allow for causal conclusions, they highlight the need to also consider *discrimination experiences* from both groups when studying the acculturation of immigrants. Our model suggests that when immigrants experience discrimination from both groups, this may lead to withdrawal from both cultural spheres and hence to marginalization, which often is associated with the worst health outcomes [4, 5].

Interestingly, the skin tone of Africans was unrelated to the discrimination they experienced. The fact that skin tone was unrelated to discrimination by Whites suggests an absence of a “pigmentocracy” in Norway, which makes sense as the country never was a colonial power so that colorism may be less engrained in society. By contrast, it suggests that the skin-tone discrimination that Africans experience by Whites in Norway is rather based on their racial group membership (i.e., being African and not White as the majority). Such a reasoning would be consistent with research showing that, for instance, individuals with each one White American and African American parent, who typically have lighter skin tone, are considered to be part of the lower status ethnic group (i.e., in this case the African American group) [45, 46]. While we were interested in the role of skin-tone discrimination, it is hence possible that the discrimination measure in our research simply functioned as a proxy for discrimination experiences based on one’s racial group membership more broadly. Future research following up on this study may therefore also include measures of discrimination based on one’s culture, ethnicity/race or religion to disentangle the role of the different types of discrimination and to establish the unique role of skin-tone discrimination. Although our test of interrater reliability suggested that our visual analogue scale was a reliable measure of participants’ skin-tone, such future research may also employ fully objective instruments such as a spectrometer.

The fact that the skin tone of participants was also unrelated to discrimination by other Africans is trickier to interpret. Previous research in the U.S. suggests that African Americans experience discrimination by their ethnic peers when they are “too dark” or “too light” [16], but no support for such a quadratic relationship was found in the present study. Addressing one methodological limitation of the present research may help elucidating this relationship: We asked participants to indicate the degree to which they felt that “African people” treat them better or worse due to their skin tone. The vague way this group was defined makes it difficult to know whether participants thought about their own ethnic group, members of other African groups, or average members of a superordinate African category (e.g., prototypical Africans) when completing the question. Still, the negative relationship between this form of discrimination and heritage culture orientation suggests that participants at least to some extent thought about their own ethnic groups when completing the measure.

Relatedly, our sample was ethnically heterogeneous, consisting of participants from a large variety of African countries. Hence, it remains possible that some form of colorism is still present within some African communities living in Norway. While the size of our sample did not allow for an adequately powered test of the relations of interest in each sub-group, it is still possible that skin color matters for the degree of in-group discrimination that African immigrants from certain ethnic groups experience in Norway.

To conclude, the present study demonstrates that both the experience of being discriminated by members of the dominant societal group and members of one’s own broader ethnic group relates to immigrants’ acculturation. While discrimination by the dominant group appears to be related to less engagement in the host culture, discrimination by ethnic peers is related to less engagement in one’s own ethnic culture.
